# Protests in Hong Kong (2019–2020): a Perspective Based on Quality of Life and Well-Being

**DOI:** 10.1007/s11482-020-09825-2

**Published:** 2020-03-13

**Authors:** Daniel T. L. Shek

**Affiliations:** Department of Applied Social Sciences, The Hong Kong Polytechnic University, Kowloon, Hong Kong

## Abstract

Triggered by the Fugitive Offenders and Mutual Legal Assistance in Criminal Matters Legislation (Amendment) Bill 2019 in Hong Kong (Extradition Bill), many protests have taken place in Hong Kong in 2019–2020. Using a perspective based on quality of life and well-being in different ecological systems, it is argued that the process of introducing the Bill is the “heat” which has ignited the “fuels” represented by 12 pre-existing and new issues in quality of life and well-being. These issues included distrust in the Central Government, lack of national identity, political dissatisfaction, economic strains, mental health threats, drop in family quality of life, lack of life skills education, lack of evidence-based national education in the formal curriculum, slow response of the Government, and alleged excessive use of force by the police. The fire has been intensified by “four strong winds”, including disinformation and misinformation, anonymity of the protesters, public support for the students, and support given by parties outside Hong Kong. Possible solutions in terms of promotion of quality of life and well-being with reference to the fire triangle are discussed.

## Protests in Hong Kong (2019–2020)

In February 2019, the Government of the Special Administrative Region, P.R.C. planned to introduce the Fugitive Offenders and Mutual Legal Assistance in Criminal Matters Legislation (Amendment) Bill 2019 (Legislative Council 2019). The main focus of the “Extradition Bill” is to regulate transfers of criminal fugitives who are wanted in different parts of China, including Taiwan, Mainland China, and Macau. This Bill constitutes a drastic change because existing laws enacted before the handover in July 1997 prohibit extradition to Mainland China. The Government proposed the Bill in February 2019 with public consultation from 12 February to 4 March 2019. There were roughly 3000 and 1400 submissions supporting and opposing the Bill, respectively (Tso 2019).

The proposed Bill has triggered a very strong reaction in Hong Kong, including numerous demonstrations, vandalism, and occupation of two universities in Hong Kong (The Chinese University of Hong Kong and The Hong Kong Polytechnic University). Starting from June 2019, the “social event” or “social unrest” continued into February 2020. Because of the novel coronavirus (COVID-19), the public attention has turned to the epidemic in mid-late January 2020. As such, the unrest appeared to “die down” a bit, although protesters wearing black clothes and face masks (and gas masks) still protested against the Government for setting up quarantine camps and clinics. Besides, the new unions of health professionals supported to strike in public hospitals during the epidemic because their demands were not fully addressed by the Government.

The social unrest brought forth by the Extradition Bill is unprecedented in terms of the duration of the movement, number of protesters, number of people being arrested, and the massive vandalism (such as damage of the Mass Transit Railway), violence of the protesters (such as around 4000 petroleum bombs found in the campus of The Hong Kong Polytechnic University after its preoccupation by the protesters) and alleged excessive force used by the police (such as firing of more than 16,000 canisters of tear gas and 10,000 rubber bullets). A close examination of the social movement shows that it has seven unique features.

First, many social unrests are triggered by economic factors, such as a rise in fuel price and high cost of living in the Yellow Vests Movement in France in 2018 and a rise in transport fare in Chile in 2019. Surprisingly, the social unrest occurred when Hong Kong had almost full employment in 2018 and excellent performance in social progress according to some international social indicators research. For example, amongst the 189 places under study, Hong Kong ranked fourth (4th) in the Human Development Index in 2018, which was the same rank as Germany (United Nation Development Program 2019). In the Human Freedom Index (Vásquez and Porčnik 2019), Hong Kong ranked third (3rd) out of 162 countries and places.

Second, people from different sectors and social classes join together in the protests, including professionals (e.g., medical doctors, allied health professionals, teachers, social workers, and information technology professionals) and middle-class people. For example, a young female designer graduated with First Class Honor was recently sentenced to imprisonment because of possession of petrol bombs. Third, many university students and high school students were actively involved in the social event. As university students have historically been involved in social movements (such as protests against the Vietnam War), it is not surprising to see their participation in this social event. Surprisingly, many high school students have also participated in the movement. According to the Commissioner of Police, 7019 people had been arrested by January 2020 (with around 40% being students) and the number of students arrested since September 2019 had increased (RTHK Radio 1 2020).

Fourth, except those organizers applying for approval for protests, no organization assumes responsibility for the social event. For many protests and strikes, it was claimed that such activities were decided and carried out by the “netizens” without the orchestration of well-structured organizations, with the decisions made on the website (Purbrick 2019). However, as many protests and strikes were well-coordinated in terms of timing and resources, it is difficult to believe that the whole movement is entirely voluntary and orchestrated solely by amateurs. For example, there are well-designed training and operation manuals for protests and strikes which can be easily downloaded from the Internet.

Fifth, vandalism has been extensive in the social event. These included damage of 740 sets of traffic lights, 52.8 km of railings along the road, and 21,800 square meters of paving blocks (Yau 2020) which require some HK$65 million to repair. Besides, there was damage of 85 Mass Transit Railway stations and 68 Light Railway stations, assault and doxing people holding contrary political views, and damage of shops owned by those who did not support the social movement (RTHK Radio 1 2020). Perhaps the most striking examples are the damage of the Legislative Council building on July 1, 2019 and the occupation of The Chinese University of Hong Kong and The Hong Kong Polytechnic University in November 2019. Besides, the use of foul language and physical violence to informally “settle” interpersonal conflicts was not uncommon in the movement.

Sixth, despite the great extent of violence and vandalism, public condemnation of such behavior was not strong. Some people even show support for the use of violence in the social movement. For example, a survey showed that roughly 20% of the respondents supported the use of violence as a tactic and more than half of the pro-democracy respondents endorsed the use of laser pointers against the police (Sum 2019).

Finally, the social event is highly political in nature. Although its origin was to protest against the Extradition Bill, it has gradually evolved into a movement with the slogan of “Five demands, not one less”. These five demands include “full withdrawal of the extradition bill”, “retracting the classification of protesters as ‘rioters’”, “amnesty for arrested protesters”, “an independent commission of inquiry into alleged police brutality”, and “dual universal suffrage, meaning for both the Legislative Council and the Chief Executive” (Wong 2019b). Besides, the slogan of “Liberate Hong Kong, Revolution of our time” emerged as the social event moved on. During the social movement, protesters have placed advertisements in foreign newspapers and waved US and UK flags during the protests, appealing for support from foreign Governments.

## Impairment of Quality of Life and Well-Being

Obviously, quality of life has been much hampered in the social movement. First, the economy has slowed down because of the social unrest. For example, the number of tourists has dropped by 56% (Cheng 2019) and the unemployment rate has been climbing in the past few months. Second, protests have created much disturbance to the ordinary lives of people. For example, during the occupation of The Chinese University of Hong Kong, the East Railway system service was much disturbed and the main highway joining the eastern part of the New Territories was blocked. The Cross-Harbor Tunnel was also out of service during the occupation of The Hong Kong Polytechnic University by the protesters. As a result, workers had to take leave involuntarily or spent much longer traveling time.

Third, the social movement has been divisive and polarized for people with different political views. For those who support the protesters and five demands, they are called “yellow ribbons”. On the other hand, those who do not support the protesters, they are called “blue ribbons”. In many families, social groups, and work settings, people have become alienated and have conflicts with each other (Chow 2019).

Finally, the social movement has created well-being problems in people of Hong Kong. Based on a large community sample, Ni et al. (2020) reported that the prevalence of probable depression was 11.2% in 2019 which was much higher than that during 2009–14 (1.9%) and 6.5% after the Occupy Central Movement in 2017. Besides, the estimated PTSD prevalence rate was 12.8%. Mogul (2019) also pointed out that PTSD symptoms were common in the protesters. Ng (2020) used the term “mental health tsunami” to describe the situation which was brought forth by the repeated and direct involvement in violent conflicts between protesters and police, exposure to violence, disintegration of families and friends, and the declining economic conditions.

## Why the Fire Broke out? Quality of Life and Well-Being Issues as Fuels

We can use “fire” to represent the social event which has lasted for roughly 8 months. Obviously, the fundamental question is why the fire broke out. In view of the highly political nature of the social movement, there are views suggesting that the event was initiated and orchestrated by forces outside Hong Kong. Such views are primarily driven by the notion of “color revolution” which has occurred in places like Tunisia and Ukraine. In addition to Western influences, Taiwan has been suspected to play a strong role in the social movement. The reasoning is that by highlighting the message that the “one country, two systems” arrangement does not work, the President of Taiwan (Tsai Ing Wen) would get more support in the presidential election in 2020. Although these conspiracy conjectures make sense on the surface, we need empirical evidence that cannot be easily substantiated. Also, conspiracy theories alone do not give full attention to the psychology of the protesters and they cannot explain the perpetual involvement of young people as well as the determination of some of them to die for the sake of producing political changes in Hong Kong.

Unfortunately, not many systematic investigations have been conducted to explore the genesis of the social movement. A pioneer paper on the protests in Hong Kong in 2019 was written by Purbrick (2019) who argued that in addition to errors in police operations, housing, poverty, and governance are three key factors underlying the social event. In this paper, we attempt to understand the social movement from the perspective of quality of life and well-being. The main thesis is that the social unrest exists because there are threats to and issues in quality of life and well-being in Hong Kong.

From an individual perspective, quality of life is a multi-dimensional construct. According to Felce and Perry (1995), overall quality of life is a function of three factors, including objective life conditions, including physical well-being (e.g., personal safety), material well-being (e.g., security), social well-being (e.g., acceptance and support), emotional well-being (e.g., respect) and development and activity (e.g., choice and control), subjective feelings of the objective life conditions and personal values and aspirations. Obviously, threats to quality of life in these domains would arouse fear and distress for an individual. From a societal perspective, Organization for Economic Co-operation and Development (OECD 2011) proposed the following indicators of human well-being: health status, work and life balance, education and skills, social connections, civic engagement and governance, environmental quality, personal security, and subjective well-being. Again, threats in well-being in these domains, particularly in the areas of governance and personal security, would lead to strong reactions from people in the society.

According to the ecological perspective, there are different ecological systems governing human development where individuals experience well-being (or ill-being) in different systems. These include the personal system (e.g., threat to freedom and finding life meaning through involvement in a “revolution”), interpersonal system (e.g., peer influence and bonding amongst peer protesters), family system (e.g., lack of family warmth), social system (e.g., sensational social media influence), and political system (e.g., lack of trust in the Government and support for protesters from bodies outside Hong Kong). By examining the quality of life and well-being in different systems, we can get some clues on the related deficiencies and threats in quality of life which have shaped the development of the social event in Hong Kong in 2019–2020.

## The Fire Triangle: Fuels, Heat, and Oxygen

According to the Fire Triangle, there are three basic elements of fire - fuels, heat, and oxygen. In this paper, we treat the quality of life and well-being issues as “fuels”, the Extradition Bill as “heat”, and some contextual influences (such as support from the general public for young people’s involvement in protests) as “oxygen”. There are ten pre-existing fuels and two new fuels for the “fire” as follows:

- *Pre-existing Fuel No. 1: Distrust in the Central Government*

Hong Kong had been a British Colony from 1841 to June 30, 1997. Although Hong Kong physically returned to China on July 1, 1997, it has been difficult for Hong Kong people to develop a high level of trust in the Beijing Government for two reasons. First, people tend to fixate on the unfortunate history of the Cultural Revolution and June 4th incident of 1989. Second, as corruption in Mainland China has been a thorny problem since the opening of China in the late 1970s, governance in China has been seen in a negative light by Hong Kong people. Empirically, studies showed that the level of trust in the Beijing Government has been fluctuating with roughly 47.7% of the respondents having no trust in the first 6 months of 2019 (Public Opinion Poll, The University of Hong Kong 2019b). Lack of trust in the Central Government clearly suggests political uncertainty which constitutes a threat to the political well-being of Hong Kong people.

- *Pre-existing Fuel No.2: Weak identification with the Chinese national identity*

Studies have shown that 75% of the young respondents regarded themselves as “Hongkonger” rather than “Chinese” (Public Opinion Poll, The University of Hong Kong 2019a). The weak national identity can be attributed to the vast differences in cultural and social background between Hong Kong and Mainland China, such as views on individual freedom and governance. The negative stereotypes formed for Mainland Chinese people (e.g., poor hygiene and snobbishness) also constitute blocks to identify with the Chinese national identity. As national identity is an important aspect of self-identity, a blurred national identity is a threat to personal well-being, which suggests a sense of rootlessness.

- *Pre-existing Fuel No. 3: Dissatisfaction with the political system in Hong Kong*

Under the British Colonial rule, the Governor was not elected but appointed by the British Government (i.e., Hong Kong people had no say). Until the last decade before the handover, the Colonial Government began to introduce some political reform initiatives. The Basic Law also stipulates that there would be a progressive change in the election systems of the Chief Executive and Legislative Council members. Although the political system can be regarded as more “open” after 1997, Hong Kong people (particularly the youngsters) are not satisfied for two reasons. First, the current political system gives heavier weight to the businessmen who have been blamed to create economic and social inequalities in Hong Kong. Second, as the Government of the SAR has not been working effectively after the handover, people generally want to have more say in important decisions for Hong Kong. Obviously, dissatisfaction with the political system is a threat to political well-being (OECD 2011).

One related factor that should be considered is the ideals of young people. In the good old days, the formula for youth development is to motivate young people to succeed in academic study (entrance to a Government-funded university and study in a professional programme), get a job with a handsome salary and establish a happy family. However, for the Generation Z (i.e., commonly refers to young people born in the late 1990s and early 2000s), they are more autonomous, technology-oriented and more concerned about social issues (Dolot 2018; Gaidhani et al. 2019). In other words, putting the material good life as the “carrot” does not really work for students of Generation Z because material possession may not be their primary concern.

- *Pre-existing Fuel No. 4: Economic strains (poverty, high housing price and high cost of living)*

Although Hong Kong enjoyed almost full employment as well as high GPD per capita in early 2019, wealth distribution has been a persistent problem: roughly one in four adolescents grow up in poor families; the Gini coefficient is disturbingly high (Oxfam 2018); there were around 1.4 million poor people with 612,900 poor households in 2018 (Government of the Hong Kong SAR 2019). Assuming 1% of the poor people were dissatisfied young people, it means around 14,000 young people were on the street protesting against the Government. Research has showed that poverty is a risk factor affecting the quality of family life and individual well-being, which would in turn undermine the healthy development of adolescents and cause problems such as the development of externalizing behavior. It also impairs the quality of life of the Hong Kong society.

Historically speaking, housing has been a thorny problem in Hong Kong. Unfortunately, the problem has been much aggravated after the handover back to China. According to some surveys, the housing price in Hong Kong and the cost of living were the highest in the world (Arcibal 2020). There are three consequences of this situation. First, young people would find it difficult to get married, hence creating much frustration in youngsters. For some of the married young couples, they may be forced to stay in sub-divided flats that have security and hygiene problems. Second, parents are expected to help children (as reflected in the saying of “the success of young people depends on the hard work of the father”) which creates much intergenerational conflict in the family. Finally, the rocket high housing price creates a sense of hopelessness in young people because it would be a heavy burden even for young professionals to buy a decent flat. The high housing price obviously triggers much negative emotions in young people. With the Extradition Bill, it is a good opportunity for them to air out their anger and hopelessness as well as a desire for “mutual destruction” (i.e., let us have nothing together). The high housing price is an obvious threat to physical well-being (shelter), psychological well-being (hopelessness), family well-being (conflict and tension within the family), social well-being (rich-poor divide), and political well-being (hatred for the Government for its ineffective housing policies).

- *Pre-existing Fuel No. 5: Lack of upward mobility*

Although there was almost full employment in 2018, youth employment has been an issue of concern for many years (Government of the Hong Kong SAR 2019). With the introduction of more self-financed sub-degree and degree programs, many graduates are not able to move up the social ladder because the real income for university graduates has been quite stagnant since the handover (New Century Forum and New Youth Forum 2015). Again, lack of upward social mobility triggers negative emotions in young people which eventually promotes a sense of hopelessness in young people (Shek and Siu 2019b). This also explains why young people have psychological resistance to return to China because their lives have not improved much after the handover. It is also why some young people waved the British flag during demonstrations which are clearly a sign of remembering the “good old days” for university students under the British rule. Obviously, the lack of opportunity for Hong Kong young people to have upward mobility is a serious threat to individual well-being and societal quality of life. Nevertheless, young people are commonly not aware of the fact that the lack of social mobility also exists in many developed countries in the world and there was also much inequality under the British rule.

- *Pre-Existing Fuel No. 6: Morbid emphasis on academic excellence*

Under the influence of the Chinese culture, Hong Kong emphasizes strongly on academic excellence and achievement, with success commonly defined in terms of good grades in public examinations and earning a lot of money. Such social mentalities have three consequences. First, striving for academic excellence can be very stressful for young people which impairs their personal well-being. One consequence is that young people are prone to develop internalizing behavior such as depression and suicide. In fact, the appeal for “mutual destruction” can be regarded as a manifestation of mass internalizing behavior. Second, the exam-oriented system naturally creates “losers” in young people. As only 18% of high school graduates can get Government-funded university places, the number of “losers” created every year is quite substantial. Third, it would be difficult for students to find authentic life meaning in study except “getting good grades”. Most of the time, young people in Hong Kong have “foreclosure” identity according to the psychosocial theory of Erik Erikson (i.e., commitment without crisis). Hence, when young people face the slogan of “Liberate Hong Kong, Revolution of the time”, the social movement gives them a noble and romantic life meaning which can be easily incorporated in their identity. In short, the morbid emphasis on academic excellence undermines the academic and personal well-being of students which can be easily filled by some heroic and grand ideals such as revolutionize Hong Kong to make the tomorrow better.

- *Pre-Existing Fuel No. 7: Psychosocial stressors and mental health issues*

Young people in Hong Kong face many psychosocial stresses, including academic stress, low income, high property price, long working hours, and a small living environment. Shek and Siu (2019b) argued that the developmental context for Hong Kong adolescents is “unhappy”, including unhealthy values, de-emphasis of holistic youth development, rise in hopelessness but drop in life satisfaction, emphasis on academic excellence but de-emphasis on academic quality of life, poverty, parenting issues, and drop in family well-being. Obviously, stresses and risk factors in adolescent development can easily be translated into poor mental health amongst young people. There are research findings showing that adolescent hopelessness rises but life satisfaction drops in adolescent years (Shek and Liang 2018). At the same time, their academic stress increases but their perceived support from school decreases (Shek and Chai 2019). In other words, the well-being of adolescents in Hong Kong is at risk. In the special issue edited by Shek and Siu (2019b), the papers show that mental health is a growing concern in young people in Hong Kong. There are also findings suggesting that mental health problems in university students are prevalent (Lo et al. 2018, 2019). In other words, young people with poor well-being are emotionally charged time bombs waiting to be detonated. Finally, students with special educational needs may be a factor that should not be overlooked. When we examine the slogans of the protesters, it is not uncommon to see that there are many incorrectly written Chinese characters. There are two possible explanations – either the protesters are poorly educated or they are dyslexic who are commonly having difficulties in writing Chinese characters. It is noteworthy that students with special education needs (e.g., those with autistic features or dyslexic) are stubborn in their views.

- *Pre-existing Fuel No. 8: Disorganization of Hong Kong families*

There are several disturbing developments of families in Hong Kong, including rising divorce and remarriage rates, rising cross-border marriages, worrying child abuse rates, growing number of parents who are not Hong Kong residents, growing cross-border workers, long working hours, and aging population. These problems negatively affect young people who experience family alienation and conflicts arising from unfavorable family circumstances. Obviously, the social event constitutes an excellent opportunity for them to feel the warmth amongst the “comrades” and have deep sharing and mutual concerns amongst the participants who may not have such warm experience before. In other words, low family quality of life is a strong precursor for active and romantic participation in the social movement. Nevertheless, there are also reports saying that some parents actually encourage their kids to actively participate in the movement, including engaging in violent behavior.

- *Pre-existing Fuel No. 9: Lack of life skills education for adolescents*

Although young people face many psychosocial stresses and challenges, there is weak systematic life skills education for adolescents. In many countries, social-emotional learning, soft skills and psychosocial competence, including the promotion of self-understanding, social understanding, interpersonal competence, responsible decision making, and self-management skills are strongly promoted. Although critical thinking is emphasized in Liberal Studies under the new high school curriculum in Hong Kong, it is argued that students actually learn “criticism mentality” instead of “critical thinking”.

In a series of cross-sectional and longitudinal studies, Shek et al. (2019) showed that while different stakeholders endorsed the importance of life skills education in the formal curriculum, they perceived that life skills education in the formal curriculum was insufficient and life skills development in adolescents was incomplete. Without such systematic education, the personal well-being of young people in Hong Kong cannot be adequately protected (Shek and Siu 2019a). The neglect of soft skills education for young people in Hong Kong means that they do not possess adequate social competence skills to negotiate with other people or resolve conflicts, which are much needed. As intense anger and hatred are involved in the social event, learning how to empathize (look at things from others’ perspective), manage one’s and others’ emotion (emotional quotient), forgive (write off emotional feelings and debts), and re-conciliate (re-build new relationships and move on) are important tasks for adolescents. These life skills are very important because there are studies showing that Hong Kong adolescents showed narcissistic behavior (Leung 2013) and positive youth development attributes negatively predicted the use of foul language in adolescents (Shek and Lin 2017).

- *Pre-Existing Fuel No. 10: Unsystematic and uncoordinated civic and national education*

Although there is an area on moral, civic and national education in the formal curriculum, the policy and scope of the national education curriculum are unsystematic and uncoordinated. In a study comparing related moral, character and citizenship education in Chinese societies (Hong Kong, mainland China, and Taiwan) and non-Chinese societies (Singapore, UK, and USA), Shek and Leung (2018) identified several problems in this area, including absence of comprehensive planning and policy development, blurred concepts and lack of focus on holistic student development, lack of emphasis of moral and character education, problem of “penetrative” approach, absence of formal curriculum materials, problematic operational strategies, and lack of evaluation. Besides, while national education is undertaken by the Education Bureau (formal school curriculum), the Home Affairs Bureau (Committee on the Promotion of Civic Education), and the Labor and Welfare Bureau (Youth Section in the Social Welfare Department), there is little coordinated effort amongst the different bureaus. The lack of related education suggests that the personal well-being of young people in terms of moral competence cannot develop in a healthy manner.

Obviously, there are two difficulties in implementing moral education in Hong Kong. The first one is “what” should be covered. If the coverage covers Chinese history in the past century, students can learn more about what happened in China, particularly the exploitation under Western Imperialism. However, as history is multifaceted, how to interpret historical facts is a thorny issue. The second issue is “how” to assess the outcomes. While an increase in knowledge is easy to demonstrate, positive change in attitude and behavior may not be easy to assess objectively. In view of the sensitive nature of Chinese History, some schools simply cut the subject under the new education reform. Under these circumstances, it is not surprising to note that many young people are not familiar with modern Chinese history and geography of China.

- *New Fuel (No. 1): Slow and ineffective responses of the Government*

The responses of the Government to the protests since June 2019 have been regarded to be slow and ineffective. Besides condemning violence and vandalism, the Government has relied primarily on the police to deal with the protests. The attempt to have dialogues with the public also does not appear to be very successful. Most important of all, although the Bill has triggered such a huge social event, no senior Government official steps down. This is very interesting because in similar situations in other countries, some senior Government officials would have stepped down to take political responsibility. Again, this reflects the threat of political well-being in governance which intensifies public worry, anger, and frustration.

Of course, in understanding the responses of the Government, two points must be noted. First, in view of the unprecedented nature and the extent of the protests, it is not easy to handle. As pointed out by the Prime Minister of Singapore (Lee Hsien Loong), Singapore would be “finished” if similar protests happened in Singapore (Sim 2019). Second, dissatisfaction with the Government has been a common theme in protests which intensifies the protests. For example, the Commission of Inquiry (1967) concluded that “a recent tendency – not only in Hong Kong – to ascribe all the failings of the community to errors by the administration and to make greater demands upon it tends not only to enhance discontent but to exaggerate their extent” (p.129).

- *New Fuel (No. 2): Alleged police violence and inaction*

There are numerous and serious allegations that the police used excessive force, such as in the protests held on June 12, July 14 and August 31, 2019 (Purbrick 2019). On the other hand, the police was criticized as doing nothing when people in Yuen Long were attacked by those who did not support the protesters on July 21, 2019. Obviously, such allegations are great threats to the personal well-being of the protesters and the social well-being of Hong Kong. On the one hand, some videos in the news reports and the Internet strongly suggest that excessive force might have been used by the police. While some of the related complaints are still under the investigation of the Independent Police Complaints Council (IPCC), the mechanism does not earn the trust of Hong Kong people for two reasons. First, some IPCC members were appointed by the Government, which means that their independence is doubtful. Second, many protesters experiencing excessive force by the police do not complain because they have the fear that they will be prosecuted for involvement in riots. On the other hand, it should be noted that “innocence before proven guilty” is the cornerstone of the Common Law and the establishment of police violence and brutality requires evidence beyond reasonable doubt. Besides, objectivity of some videos uploaded to the Internet is not clear. In addition, it would not be objective if we ignore the fact that public perceptions of the police had been very good before the social event and the Hong Kong Police ranked very high in terms of professional service in international surveys. For example, in the Human Freedom Index (Vásquez and Porčnik 2019), Hong Kong police ranked sixth (6th) under the indicator of “reliability of police”. In the Legatum Prosperity Index (2019), Hong Kong police ranked fourth (4th) out of 167 countries and regions under the indicator of “safety and security”. Similarly, Hong Kong police ranked fourth (4th) under “order and security” in the World Justice Project (2019). Of course, having an excellent record does not necessarily mean that the alleged police issues do not exist. However, we also need expert views based on credible professionals (e.g., those who have expertise in police operations) using credible evidence from credible sources to make objective and fair judgments.

## Heat (Extradition Bill)

There are several arrangements that create anxiety and threats for Hong Kong people. First, it extends the scope of extradition to cover Mainland China. With the proposed changes, Hong Kong people who have committed certain crimes in China could be transferred to Mainland China. Second, there were several rounds of revisions in the process, thus giving people a sense that the whole package has not been well-conceived, and the changes were made to address the concerns of the businesspeople only. Third, the proposed safeguards are considered not adequate by the public. Finally, the consultation period was too short. Some people queried that the consultation was too short for an issue which had not been resolved within 22 years after the handover. At the same time, the buy-in work was not enough, and consultation was not extensive. In particular, no specific strategies were used to address the concerns of young people, particularly via social media. Besides, the publicity work of the Government was neither creative nor innovative.

Finally, the Government’s non-sensitivity about the public reaction to the Bill greatly intensified the fear. Since the proposal was published, there had been numerous reservations voiced by different sectors of the society, including businessmen, lawyers, and barristers. However, the Government did not feel the pulse of the community. Also, despite the fact that many people joined the protest held on June 9, the Government still decided to move to the second reading debate on June 12, 2019. Such insensitivity to public reaction eventually triggered the fire.

## The Four Strong Winds

For the political fire on the Extradition Bill to take place, besides fuels (quality and life and well-being issues) and heat (worries that the Bill had created and the public sentiment it had aroused), oxygen is a very important concern. In the social movement, several sources of wind have provided much “oxygen” for the social event.

- *Wind No. 1: Misinformation and Disinformation*

While misinformation refers to inaccurate information, disinformation refers to the deliberate dissemination of false information. In the social movement, there are numerous instances of misinformation. For example, for the number of protesters, it was claimed that there were 1 million and 2 million people joining the demonstrations in June 2019. However, while it cannot be denied that many Hong Kong people joined the protests, the figures quoted by the organizer were doubted by CNN (Mezzofiore 2019). Concerning the occupation of the Legislative Council Building on July 1, 2019, the police issued a warning at around 10:20 pm condemning the action of the protesters and giving the final warning for them to disperse. However, the watch of the Chief Superintendent had been altered to 5 pm (Wong 2019a) in the video which suggests that the police had set a trap for the protesters. Another example is the news reported by Da Kung Pao on the assault on a Legislative Councilor (Ho Kwan Yiu). While the news was released at noon on November 6, 2019, the time of release was amended to be 19:54 on November 5, 2019 (Global Times 2019). Based on the amended news, it was claimed that the assault was self-directed by Ho.

- *Wind No. 2: Anonymity*

In the initial stage of the protests, many protesters wore surgical masks. In the late stage of the protesters, some protesters wore gas masks and covered their whole face. While it is understandable that gas masks protect the protesters from tear gas, keeping one’s identity anonymous actually intensified the scale of violence and vandalism because the fear of being identified would be minimized. Besides, communication in social media on protests is also anonymous, hence facilitating the planning and implementation of the protests and vandalism. As it is well-documented in social psychology that people with anonymous identity would be more likely to engage in violent behavior (Zimbardo 1969), anonymity has supplied much “oxygen” to the intensity and duration of the social event.

- *Wind 3: Public Support for the Protesters*

Many people have shown support for the protesters (particularly the students) for several reasons. First, it is commonly believed that the public should give more allowance to students who are just “kids”. Second, some adults have the fear that the Bill would break the “firewall” between Hong Kong and China. Third, some people believe that the students are doing what they have not done, such as a fight for democracy for Hong Kong. Fourth, many people are angry at the slow and non-responsive responses of the Government. Fifth, many people support the prevailing but toxic beliefs that “disobey the law to get justice is acceptable” and “violence is sometimes necessary under certain circumstances”. Finally, many people believe that violence of the protesters is justified because police have used excessive force and there is police brutality. There is also public support in terms of finance. For example, around HK$70 million (US$9 million) related to the Sparkle Alliance was frozen by the police (Mok et al. 2019). The public’s support for the protesters (including many professional associations) and not “cutting the mattress” with the violent protesters is definitely a strong reinforcement for the protesters.

- *Wind No. 4: Overseas Support*

In the social event, many foreign political leaders have shown support for the protesters. Unfortunately, very few of them touch upon the issues of violence and vandalism. Such supportive gestures have created the false impression that the movement (including violence and vandalism) is reasonable, sacred and just. Material support from Taiwan in the form of gas masks was also reported (Sui 2019).

## Way Forward

It is apparent that there are many deep-seated quality of life issues behind the social movement. As such, the solution lies in properly addressing these quality of life threats and issues. Primarily, it is important to cut or eliminate the pre-existing fuels as follows:

1. *Trust in the Beijing Government*: It is important to build up trust in the Beijing Central Government. In social psychological literature, the contact hypothesis suggests that contact is very important to reduce prejudice (Pettigrew and Tropp 2005). Hence, increased contact to understand Mainland China will be helpful. Looking at the bright and dark sides of China (as these two aspects exist in all countries) and appreciating the progress of China in the past four decades such as poverty alleviation for around 850 million people according to the World Bank (He 2019) and acknowledgment of social problems (such as corruption) would be important.
2. *Nurturance of Chinese national identity:* It takes time to nurture because one has to “fall in love” and take pride in being Chinese. Systematic education at the community, family, and school contexts are indispensable so that young people can develop a sense of shared identity which further strengthens their positive identity.
3. *Political well-being:* This is tough because the change in the political system will not take place overnight. In addition, as Hong Kong is an international financial center, it is not easy to reduce the influence of business corporations and related interest groups. However, given the recent overwhelming victory of the Pan-Democratic camp in the District Council election in November 2019, young people who wish to change can still play an active role in the future Legislative Council and the Chief Executive elections. In any case, everybody has to learn that in an open and civilized society, we have to respect the views of the majority.
4. *Poverty alleviation and solving the housing problem*: It is a high time to re-visit the issue of poverty in Hong Kong which also exists in many countries in the world. Besides the work of the Commission on Poverty, every sector has to re-think about their role. In addition to financial capital, building human capital, family capital and social capital is also important to strengthen the developmental assets of poor adolescents, which can help them escape from the trap of inter-generational poverty. As housing implies physical security which shapes hope, this is the top priority issue to be addressed.
5. *Upward mobility*: This is not easy to solve this issue because it is a global issue that is not specific to Hong Kong. However, as many young people do not want to join the manual labor force which can give a reasonable salary, there is a need to change the community culture to one that treasures different talents (i.e., not just scholastic skills) and there are different career paths for young people with different aspirations, interests, and skills.
6. *Education reform:* There is a need to deeply reflect on the purpose and nature of education so that young people can study for their interests (i.e., not just for the “rice bowl”) and multiple intelligences are emphasized. The challenge is not just for the Government, but also for parents and the Hong Kong society.
7. *Promotion of adolescent well-being:* Relevant and adequate services should be provided for young people who need mental health services. A public health approach focusing on universal, selective and indicated prevention should be used. In particular, services for students with special education needs should be stepped up. Besides, finding ways to promote hope and life satisfaction is also a priority task. In addition to changing the macro-environmental factors leading to hopelessness, evidence-based programs to cultivate resilience and hope in young people, such as the Project P.A.T.H.S. (Shek and Sun 2013; Ma et al. 2019) are indispensable.
8. *Promotion of family well-being:* Problem families are the ideal breeding grounds for adolescent externalizing behavior. Hence, there is a need to promote family resilience in a high-risk environment and to strengthen parenting, communication as well as conflict resolution skills in the family. It is noteworthy that evidence-based programs on promotion of family resilience are almost non-existent in Hong Kong.
9. *Promotion of Life skills education:* In the absence of systematic life skills, social-emotional learning skills, and psychosocial competencies training in the formal education curriculum in Hong Kong, this gap should be urgently addressed (Shek and Siu 2019a). Young people should learn that: a) “an eye for an eye makes the whole world blind”; b) the essence of democracy is respecting the views of everyone, including those who do not hold the same views; c) it is morally wrong to blackmail the Government by kidnapping the innocent public; d) besides rights and social justice, there are other virtues, including love, acceptance, and forgiveness, that are equally important; and e) while one can “criticize” the Government, one should also look at things from different perspectives in a “critical” manner.
10. *Development and implementation of systematic moral and national education*: One unfortunate observation of the social movement is that there is much hatred and deep erosion of the spirit of rule of law (such as widespread use of “doxing” and even physical violence in dealing with those who hold different views). As conflict resolution and mutual respect are not adequately covered in the current moral and national education curriculum, there is an urgent need to review and step up the related policies in Hong Kong. When implemented, policymakers should be aware of the related fears, such as the worry of brainwashing the young people. As such, practices in other countries can serve as the reference point because “international standards” will be used.

Regarding the “heat”, there are many learning points for the Government to reflect, particularly on its efficiency and responses in the whole social movement. For example, sufficient consultation time should be given to sensitive issues like the Extradition Bill. Concerning the influence of social media, there is no way to stop it unless we follow what the Spanish Government did to deal with the occupation of the airport. However, the public can learn how to critically differentiate correct information, misinformation, and disinformation, particularly information disseminated and acquired through the social media. For anonymity, while it is a protection for not being arrested, people wearing masks should understand that anonymity would unleash the dark side of human nature.

Concerning public endorsement of violence, several questions should be considered by the public: a) What is the “civilized” and “mature” form of public demonstration? b) Assuming the police has used excessive force, is vandalism the best response (i.e., “an eye for an eye”)? c) How can we promote peaceful co-existence within diversity in political views? These are obvious quality of life issues awaiting answers. Regarding alleged police violence brutality, there is a social consensus on setting up an independent panel of inquiry. However, to be fair to the police and protesters, if an independent panel of inquiry is established, it should examine violence and excessive use of force in both the protesters and police. Finally, a critical understanding of the comments made by foreign Governments and parties should be realized – whether they are genuine concerns about Hong Kong or disguised manifestations of political and/or national interest.

In conclusion, pre-existing and new quality of life and well-being threats and issues have shaped the development of the social event in the past 8 months in Hong Kong. Without understanding quality of life issues in different ecological systems (such as fear about losing freedom, lack of political well-being, growing up in a poor environment, always being a loser, living without hope, inability to forgive and re-build), it would not be possible to find workable and meaningful solutions. Once again, the case of Hong Kong demonstrates that economic development alone is not enough to promote human well-being in a society.

In the report of the Commission of Inquiry (1967) reviewing the 1966 riots, it was concluded that “we do not believe that political, economic and social frustrations were the direct cause of the 1966 riots but within the economic and social fields there are factors, to which we have drawn attention and that need to be watched, lest they provide inflammable material which would erupt into disturbance should opportunity arise in the future” (p.148). After some 53 years, it is interesting to note that some of the deep-seated quality of life and well-being issues, such as inadequate housing, over-crowding, and limited chances in life come into the scene again. The only major difference is that many educated young people are involved in the social event in 2019–2020, in contrast to the predominance of poorly educated young people in the 1966 riots.
